# Survival Following a Tricyclic Antidepressant Overdose Presenting With Serotonin Syndrome-Like Symptoms and Critical Illness Polyneuropathy: A Case Report

**DOI:** 10.7759/cureus.100671

**Published:** 2026-01-03

**Authors:** Shigehiro Nakamura, Ryuichiro Okuda, Tatsuo Takama, Yasuyuki Kakihana, Shuhei Niiyama

**Affiliations:** 1 Emergency and Critical Care Center, Kagoshima City Hospital, Kagoshima, JPN; 2 Department of Intensive Care Medicine, Kagoshima City Hospital, Kagoshima, JPN; 3 Department of Emergency and Intensive Care Medicine, Kagoshima University Graduate School of Medical and Dental Sciences, Kagoshima, JPN

**Keywords:** acute respiratory distress syndrome (ards), critical illness polyneuropathy, neurological complication, neuromuscular complications, serotonin syndrome, tricyclic antidepressant overdose

## Abstract

We report the case of a patient who ingested a lethal dose of amitriptyline, presented with serotonin syndrome-like neurological symptoms on arrival, and subsequently developed suspected critical illness polyneuropathy (CIP) during the clinical course. A 54-year-old man with a history of depression intentionally ingested approximately 2,250 mg of amitriptyline in a single dose. On arrival, he exhibited altered consciousness, hyperthermia, metabolic acidosis, and QRS prolongation, necessitating the immediate initiation of mechanical ventilation and intravascular cooling. Despite sedation, myoclonus emerged, raising suspicion of serotonin syndrome based on the presence of hyperthermia, myoclonus, and altered mental status. Clonazepam, levetiracetam, and cyproheptadine were administered, along with alkalinization therapy. During hospitalization, the patient developed aspiration pneumonia that progressed to acute respiratory distress syndrome, requiring prone positioning and prolonged mechanical ventilation. Profound respiratory muscle weakness necessitated tracheostomy. Subsequently, limb weakness and nerve conduction study findings raised suspicion for CIP. This case highlights that tricyclic antidepressant intoxication can manifest with diverse neurological and neuromuscular complications, necessitating comprehensive management from the acute through chronic phases.

## Introduction

Tricyclic antidepressants (TCAs) possess substantial cardiotoxic and central nervous system-depressant properties, and severe overdose is frequently fatal [1]. The therapeutic index of TCAs is narrow; therefore, ingestion of 10-20 mg/kg is potentially life-threatening [2-4]. We herein report a case in which a patient ingested approximately twice the lethal dose of amitriptyline and survived with intensive care management. The patient initially presented with findings resembling serotonin syndrome-a potentially life-threatening syndrome with manifestations spanning from mild adverse effects to life-threatening toxicity - and later developed suspected critical illness polyneuropathy (CIP) - a complication of critical illness marked by muscle weakness and failure to wean from the ventilator-making this a clinically significant case.

## Case presentation

**Figure 1:**
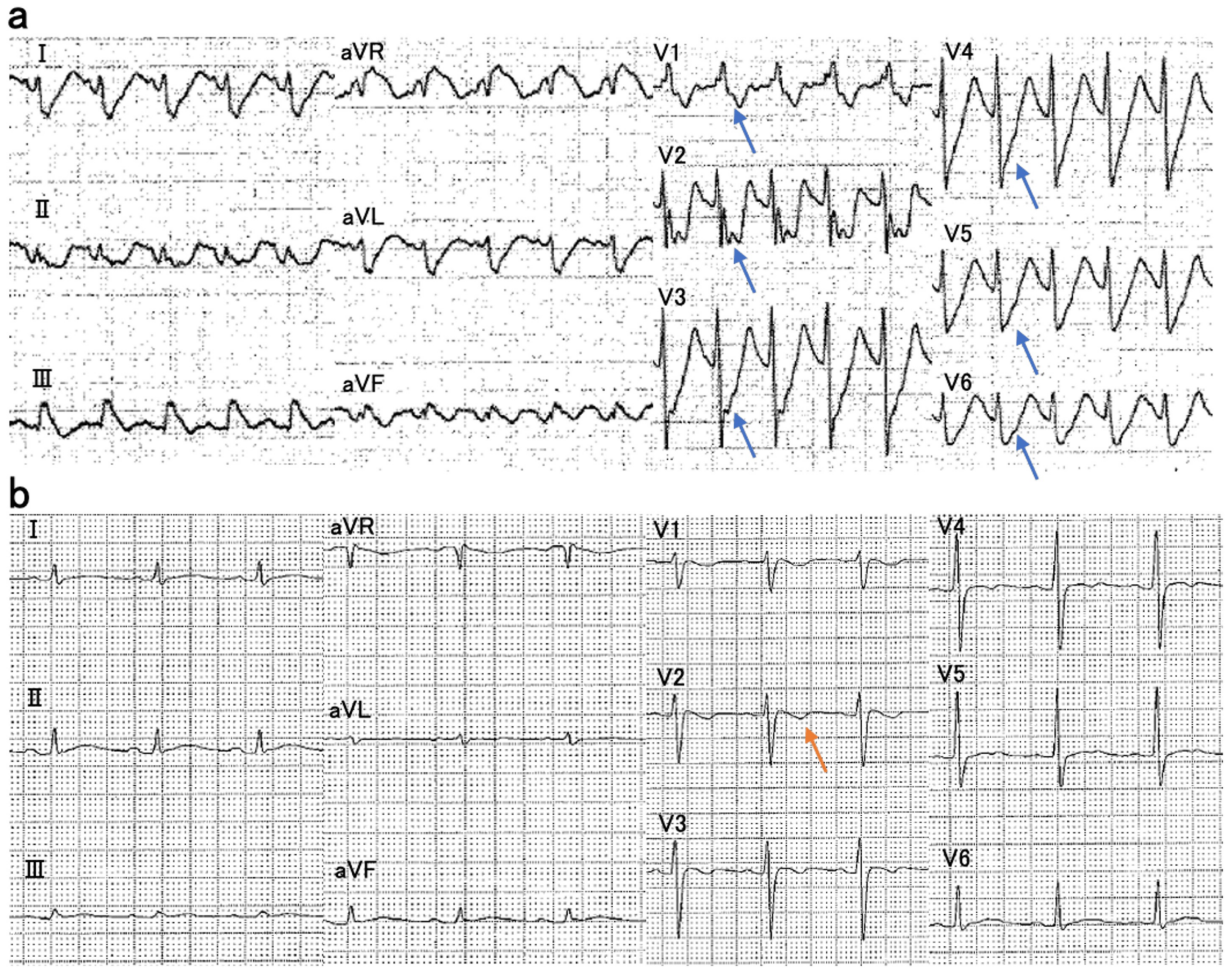
Electrocardiography changes (a) On admission: sinus tachycardia with ST depression in leads V1–V6, widened QRS complexes (154 ms), QTc prolongation (466 ms), and right-axis deviation were observed. Sinus tachycardia reflects the anticholinergic effects of TCA, and QRS widening indicates sodium channel blockade. The QRS duration of 154 ms was clinically important, and it approached the critical threshold of 160 ms, which is associated with a high risk for life-threatening ventricular arrhythmias. (b) Hospitalization day 4: sinus rhythm with a negative T wave in lead V2 was observed, and the QRS duration, QT interval, and electrical axis were within normal limits.

A 54-year-old man with a history of depression intentionally ingested an estimated 2250 mg of amitriptyline (approximately 40 mg/kg) in a suicide attempt. On arrival, he presented with impaired consciousness, and his level of consciousness was assessed using the Glasgow Coma Scale, with a score of E1V2M1 [5]. His body temperature was 42°C, pulse rate was 94 beats/min, and blood pressure was 63/49 mmHg. A 12-lead electrocardiogram revealed QT prolongation (466 ms) and marked QRS widening (154 ms) (Figure 1a).

Arterial blood gas analysis under a reservoir mask at 15 L/min showed severe metabolic acidosis, with a pH of 7.209, PaCO₂ of 45.6 mmHg, PaO₂ of 107 mmHg, HCO₃⁻ of 17.5 mmol/L, base excess of −10.0 mmol/L, glucose of 195 mg/dL, and lactate of 1.9 mmol/L. Immediate endotracheal intubation and mechanical ventilation were initiated. Surface cooling using ice packs applied to the axillae and groins, along with administration of acetaminophen (1,000 mg), failed to reduce the patient’s fever; therefore, intravascular cooling was initiated to prevent organ damage. Empiric treatment for bacterial pneumonia and suspected meningitis was immediately initiated with meropenem 2 g intravenously every 8 h (IV q8h). The cooling device was discontinued on hospitalization day 3 after his core temperature decreased to <40°C.

Despite adequate sedation, myoclonus persisted. Given the combination of hyperthermia, myoclonus, and altered mental status, serotonin syndrome was suspected according to the Hunter Serotonin Toxicity Criteria [6]. Clonazepam (1 mg/day), levetiracetam (1,000 mg/day), and cyproheptadine (24 mg/day) were initiated, along with alkalinization therapy. Sodium bicarbonate was administered as an initial bolus of 100 mEq, followed by a continuous intravenous infusion at a rate of 10 mEq/h, aiming for a serum pH of 7.50-7.55. The serum pH reached 7.53 on the same day, and the continuous infusion was discontinued on hospitalization day 8.

On hospitalization day 7, he developed aspiration pneumonia that progressed to acute respiratory distress syndrome (ARDS). His oxygenation index showed a markedly reduced PaO₂/FiO₂ (P/F) ratio of 74.6 (PaO₂ 74.6 mmHg at FiO₂ 1.0), which met the criteria for severe ARDS based on the Berlin Definition [7]. The antibiotics were changed to piperacillin/tazobactam 4.5 g intravenously every 8 h (IV q8h), and steroids (methylprednisolone 40 mg/day) were administered. Prone positioning was also performed under deep sedation and neuromuscular blockade, with rocuronium administered at 0.2 mg/kg/h. Following intensive management, his respiratory status improved considerably, with the P/F ratio reaching 300. Consequently, neuromuscular blockers and steroids were discontinued on hospitalization day 10. Despite subsequent improvement in mental status, an attempt was made to extubate him; however, re-intubation was required because of hypoxemia caused by difficulty in clearing secretions.

**Figure 2:**
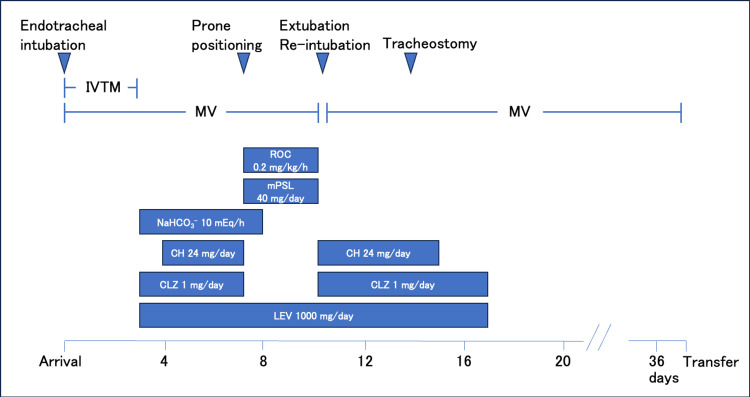
Clinical course CH: cyproheptadine; CLZ: clonazepam; IVTM: intravascular temperature management; LEV: levetiracetam; mPSL: methylprednisolone; MV: mechanical ventilation; NaHCO_3_^−^: sodium bicarbonate; ROC: rocuronium

As profound respiratory muscle weakness impeded ventilator weaning, a tracheostomy was performed on hospitalization day 14. Cyproheptadine and piperacillin/tazobactam were discontinued on hospitalization day 15, and both clonazepam and levetiracetam were stopped on hospitalization day 17 (Figure 2).

On hospitalization day 22, nerve conduction studies were performed to evaluate persistent limb weakness. The results demonstrated markedly reduced amplitudes of compound muscle action potentials and sensory nerve action potentials in the tibial nerve, suggesting the development of CIP.

His general condition gradually stabilized, and he was transferred to another facility on hospitalization day 37 for long-term management and rehabilitation.

## Discussion

TCA poisoning causes anticholinergic symptoms and sodium channel blockade, resulting in manifestations such as dry mouth, somnolence, confusion, mydriasis, tachycardia, urinary retention, and paralytic ileus [2,8]. In severe cases, patients may develop impaired consciousness, arrhythmias, and circulatory collapse [9,10]. QRS interval prolongation is an important indicator of toxicity severity; a QRS duration exceeding 100 ms is associated with an increased risk of seizures, whereas durations exceeding 160 ms are linked to a higher likelihood of ventricular arrhythmias [11]. In addition to airway protection and other supportive measures, sodium bicarbonate administration is considered the mainstay of therapy.

In the present case, the patient exhibited profound impairment of consciousness and marked QRS prolongation on arrival. However, early hemodynamic stabilization and alkalinization therapy with sodium bicarbonate prevented the development of life-threatening arrhythmias or cardiac arrest. Although lipid emulsion therapy has been reported as a potential rescue treatment for intoxication with lipophilic agents, such as TCAs, its indications, optimal dosing, and criteria for efficacy remain controversial [12]. The patient’s survival without lipid therapy further emphasizes the importance of prompt circulatory stabilization and comprehensive supportive management.

Another notable feature of this case was the presence of hyperthermia and myoclonus on admission, consistent with a serotonin syndrome-like presentation. Amitriptyline inhibits serotonin and norepinephrine reuptake, and severe overdose alone can result in serotonergic excess. Serotonin syndrome is characterized by a triad of altered mental status, autonomic instability, and neuromuscular hyperactivity-including hyperreflexia, myoclonus, and rigidity-and is commonly diagnosed using the Hunter criteria [13]. Although the patient met the criteria based on hyperthermia and myoclonus, the absence of rigidity or hyperreflexia and the unusually prolonged clinical course rendered this an atypical presentation.

Furthermore, the patient was suspected of having developed CIP during the clinical course. CIP is a distal axonal sensory-motor polyneuropathy that typically affects the limb and respiratory muscles symmetrically, and it is often more pronounced in the lower extremities, while sparing the facial muscles. Although literature suggests that TCA may cause Wallerian degeneration when used as a local anesthetic [14], a direct causal relationship between systemic TCA overdose and the observed neuromuscular dysfunction could not be established in this case. However, it is more likely that multiple factors-including hypotension, prolonged sedation, and systemic inflammation-collectively contributed to its development. CIP is a well-recognized cause of ventilator weaning failure and delayed rehabilitation, highlighting the importance of early neuromuscular evaluation and mobilization in critically ill patients [15].

Although the mortality rate of amitriptyline overdose remains high, survival has been reported when early circulatory and respiratory management is initiated [16]. This case represents a rare instance of survival despite ingestion of a dose exceeding the lethal threshold, proving the vital role of aggressive and comprehensive supportive management. Furthermore, the complex clinical course involving serotonin syndrome-like manifestations and suspected CIP reminds clinicians of the need to maintain a high index of suspicion for diverse neurological and neuromuscular complications, which is essential for prompt evaluation and management in cases of severe TCA intoxication.

## Conclusions

We report a rare case of survival following ingestion of a typically lethal dose of amitriptyline. The patient exhibited serotonin syndrome-like manifestations in the acute phase and later developed suspected CIP. In cases of TCA intoxication, sustained aggressive supportive management, regardless of the ingested dose, is crucial. Furthermore, a multidisciplinary approach is required to effectively address the complex and diverse neurological and neuromuscular sequelae observed in severe presentations. Accumulating case reports that document these prolonged recovery phases may inform future clinical guidelines, particularly in optimizing rehabilitative protocols and long-term management strategies for severe intoxication.
